# Regulation of mTORC1 by Upstream Stimuli

**DOI:** 10.3390/genes11090989

**Published:** 2020-08-25

**Authors:** Chase H. Melick, Jenna L. Jewell

**Affiliations:** 1 Department of Molecular Biology, University of Texas Southwestern Medical Center, Dallas, TX 75390, USA; chase.melick@utsouthwestern.edu; 2 Harold C. Simmons Comprehensive Cancer Center, University of Texas Southwestern Medical Center, Dallas, TX 75390, USA; 3 Hamon Center for Regenerative Science and Medicine, University of Texas Southwestern Medical Center, Dallas, TX 75390, USA

**Keywords:** mTORC1, amino acids, G-protein coupled receptors, small GTPases, kinases, phosphorylation, cell growth, metabolism, and autophagy

## Abstract

The mammalian target of rapamycin (mTOR) is an evolutionary conserved Ser/Thr protein kinase that senses multiple upstream stimuli to control cell growth, metabolism, and autophagy. mTOR is the catalytic subunit of mTOR complex 1 (mTORC1). A significant amount of research has uncovered the signaling pathways regulated by mTORC1, and the involvement of these signaling cascades in human diseases like cancer, diabetes, and ageing. Here, we review advances in mTORC1 regulation by upstream stimuli. We specifically focus on how growth factors, amino acids, G-protein coupled receptors (GPCRs), phosphorylation, and small GTPases regulate mTORC1 activity and signaling.

## 1. Introduction

In 1964, a scientific expedition ventured to Rapa Nui (also known as Easter Island) to collect soil and plants samples [1,2,3]. These samples were brought back to Canada, and rapamycin was isolated from the bacterium Streptomyces hygroscopicus in 1972. Initially, rapamycin was characterized as an antifungal agent, and further studies identified rapamycin to be an immunosuppressant. The ability of rapamycin to inhibit cell growth was discovered later. Experiments demonstrated that rapamycin formed a complex with peptidyl-prolyl cis-trans isomerase FK506-binding protein 12 (FKBP12) [4]. Through genetic screens, the target of rapamycin (TOR) was first discovered in yeast, where mutations in TOR were resistant to rapamycin [5,6,7]. Biochemical experiments in mammalian cells revealed that the rapamycin-FKBP12 complex specifically targets and inhibits the mammalian target of rapamycin (mTOR) [8,9,10]. Through affinity purification, the FKBP12-rapamycin complex was shown to bind a large molecular weight protein called mTOR (also referred to FRAP, RAFT1). Currently, rapamycin and rapamycin analogs (rapalogs) are commonly used as cancer and transplant therapeutics. Decades later, the precise mechanism of how mTOR is regulated is still being elucidated. mTOR coordinates multiple physiological processes through downstream signaling networks. In this review, we discuss the progress made in the understanding mTOR, specifically mTOR complex 1 (mTORC1) regulation by upstream stimuli.

## 2. mTOR

mTOR is an evolutionarily conserved Ser/Thr protein kinase that is classified in the phosphatidylinositide 3 kinase (PI3K)-related kinase family within the human phylogenetic kinome tree. mTOR functions as the catalytic subunit of two distinct complexes, referred to as mTORC1 and mTORC2. Rapamycin and rapalogs inhibit mTORC1 activity allosterically, while mTORC2 demonstrates short-term rapamycin insensitivity [11,12,13]. The rapamycin-FKBP12 complex binds to the FKBP12-rapamycin-binding (FRB) domain on mTOR reducing availability of the catalytic cleft, resulting in some substrates unable to access the active site. Prolonged treatment of rapamycin is thought to inhibit mTORC2 through the sequestration of mTOR in some cell types [14,15]. ATP-competitive inhibitors like Torin1 have also been developed, which directly target the catalytic site and inhibit the kinase activity of mTOR [16].

## 3. mTORC1

**Figure 1:**
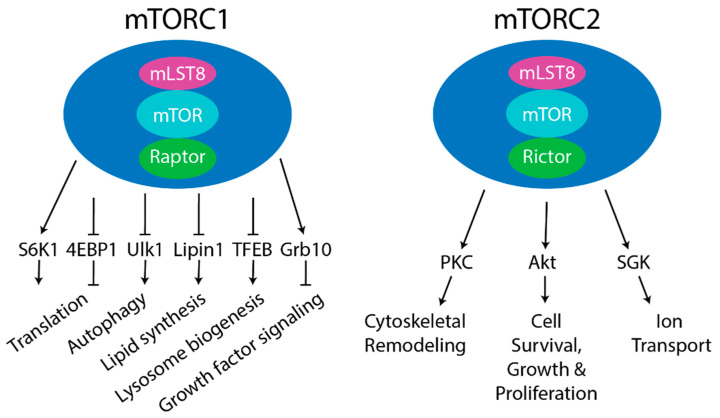
Components of mTOR complex 1 (mTORC1) and mTORC2. Left- Core components of mTORC1 are mammalian target of rapamycin (mTOR) (kinase), Raptor (substrate recognizing component), and mLST8 (positive regulator). Other reported mTORC1 components are PRAS40 (negative regulator) and DEP-domain-containing mTOR-interacting protein (DEPTOR) (negative regulator). Five main downstream pathways are shown. The phosphorylation of S6 kinase 1 (S6K1) and 4EBP1 by mTORC1 regulates protein translation. The phosphorylation of ULK1 by mTORC1 regulates autophagy. mTORC1 also regulates lipid synthesis by phosphorylating S6K1 or Lipin1 to control SREBP, lysosome biogenesis by phosphorylating TFEB, and growth factor signaling by phosphorylating Grb10. Right- Core components of mTORC2 are mTOR (kinase), Rictor (substrate recognizing component), and mLST8 (positive regulator). Other complex components include mSin1 (positive regulator), Protor1/2 (positive regulator), and DEPTOR (negative regulator). mTORC2 regulated processes include cytoskeletal remodeling by phosphorylating PKC; cell survival, growth, and proliferation by phosphorylating Akt; and ion transport by phosphorylating SGK.

mTORC1 consists of three main core components: mTOR, regulatory protein associated with mTOR (Raptor) and mammalian lethal with Sec13 protein 8 (mLST8, also referred to as GβL) (Figure 1, Left) [17,18,19]. Raptor acts as a substrate recognizing subunit that facilitates mTOR phosphorylation through the TOR signaling (TOS) motif found in some mTORC1 substrates [20,21]. Mutations in the TOS motif were shown to render mTORC1 downstream targets, such as the phosphorylation of p70 ribosomal S6 kinase 1 (S6K1) and eIF4E-binding protein 1 (4EBP1, also known as PHAS-1), insensitive to amino acid changes [22]. mLST8 is a positive regulator of mTORC1, stabilizing the association between Raptor and mTOR, and stimulating mTOR kinase activity [19]. mTORC1 contains two additional negative regulators, Proline-rich Akt substrate 40 kDa (PRAS40) [23,24,25] and DEP-domain-containing mTOR-interacting protein (DEPTOR) [26]. PRAS40 acts as a direct inhibitor of substrate binding through the interaction with Raptor, repressing mTORC1 activity [24]. PRAS40 phosphorylation by mTORC1 relieves the negative regulation, increasing mTORC1 signaling [27]. The postsynaptic density 95, discs large, zonula occludens-1 (PDZ) domain of DEPTOR directly interacts with mTOR to inhibit activity [26]. Additionally, mTOR has been shown to promote its own activity via the E3 ubiquitin ligase Skp1, Cullin1, F-box (SCF) adaptor, βTrCP, mediated degradation of DEPTOR [28,29,30].

When localized to the lysosome, mTORC1 directly interacts with and is activated by the small GTPase Ras homolog enriched in brain (Rheb) [24,31]. However, some mTORC1 mediated process, such as protein translation, presumably occur in the cytoplasm [32]. Additionally, mTORC1 has been observed in other subcellular locations such as the mitochondria [33], stress granules [34], and at the plasma membrane [35]. mTORC1 components have also been reported at multiple locations within the cell [36]. For example, mTOR and Raptor were detected in the nucleus [37]. A more complete discussion of this topic has been reviewed previously [36].

mTORC1 regulates a multitude of cellular processes, such as protein translation, autophagy, lysosome biogenesis, lipid synthesis, and growth factor signaling [38]. mTORC1 regulates translation via the phosphorylation of S6K1 at Thr 389 to activate S6K1 [17]. S6K1 then proceeds to promote translation initiation through the subsequent phosphorylation of factors such as eukaryotic translation initiation factor 4B (eIF4B) [39]. Additionally, mTORC1 phosphorylates 4EBP1 at multiple sites (Thr 37, Thr 46, Ser 65, Thr 70) to promote translation [40]. Once 4EBP1 is phosphorylated it dissociates from eIF4E, which allows the recruitment of the other translation initiation proteins eIF4G and eIF4A [41]. mTORC1 disrupts Unc-51 like autophagy activating kinase 1 (ULK1) interaction with 5′AMP-activated protein kinase (AMPK) through the phosphorylation of Ser 757 (equivalent to Ser 758 in human) on ULK1, to regulate autophagy [42]. Sterol-responsive element-binding protein (SREBP) promotes de novo lipid synthesis [43]. mTORC1 positively regulates SREBP through the phosphorylation and activation of S6K1 or through the multiple site phosphorylation and inhibition of Lipin1, another mTORC1 substrate [43,44,45]. mTORC1 negatively regulates transcription factor EB (TFEB), which promotes genes for lysosomal biogenesis and autophagy machinery at Ser 142 and Ser 211, preventing TFEB nuclear translocation [46,47,48]. Phosphorylation of growth factor receptor-bound protein 10 (Grb10) by mTORC1 at Ser 501 and Ser 503 negatively regulates growth factor signaling through IGF-1 receptor [49,50]. A more comprehensive review of mTORC1 substrates and downstream signaling pathways controlled by mTORC1 is elsewhere [38,39,51].

## 4. mTORC2

Similar to mTORC1, mTORC2 consists mTOR and mLST8. However, mTORC2 contains rapamycin insensitive companion of mTOR (Rictor) as the substrate recognizing component (Figure 1, Right) [12,13]. Additionally, mTORC2 is comprised of the negative regulator DEPTOR. mTORC2 contains mammalian stress-activated MAPK-interacting protein 1 (mSin1) [52,53,54], which is necessary for the assembly of mTORC2 on the plasma membrane [55]. Activation of mTORC2 depends on the pleckstrin homology (PH) domain of mSin1 that binds to phosphatidylinositol 3,4,5-triphosphate (PtdIns(3,4,5)P3, also referred to as PIP3) at the plasma membrane [56]. Lastly, mTORC2 consists of protein observed with Rictor 1/2 (Protor1/2, also known as PRR5) [57].

mTORC2 has been observed in multiple locations throughout the cell. Using a reporter of endogenous mTORC2 activity, a study showed mTORC2 associates with the plasma membrane, mitochondria, and on endosomal vesicles [58]. mTORC2 has also been reported to localize to the endoplasmic reticulum (ER) and ER associated membranes, such as mitochondria-associated ER membranes (MAMs) [36]. mTORC2 can regulate MAM integrity through the mTORC2 substrate Rac-α Ser/Thr-protein kinase (Akt, also known as PKB) [59]. Lastly, evidence showed mTORC2 may shuttle to the nucleus, however the function of mTORC2 in the nucleus remains unknown [37].

mTORC2 regulates physiological processes through the phosphorylation and activation of downstream substrates like the protein kinase A, G and C (AGC) family. Protein kinase C α (PKCα) at Ser 657 was the first identified substrate of mTORC2 [13]. Other PKC family members have been shown to be phosphorylated and activated by mTORC2, including PKCδ, PKCξ (Thr 560), PKCγ, and PKCε to regulate cytoskeletal remodeling and cell migration [60,61,62]. mTORC2 phosphorylates Akt on Ser 473 to promote cell survival, proliferation and growth [63]. Akt mediates these processes through the subsequent phosphorylation of substrates such as Forkhead box O1/3 (FoxO1/3a) at Thr 32 and Ser 253, glycogen synthase kinase 3 β (GSK3-β) at Ser 9, and tuberous sclerosis complex 2 (TSC2) at Ser 939 and Thr 1462 [54,64,65,66]. Lastly, through the phosphorylation of serum/glucocorticoid-regulated kinase 1 (SGK1) at Ser 422, mTORC2 controls processes like ion transport and cell growth [67]. mTORC2 will not be discussed further in this review, and mTORC2 has been reviewed elsewhere [38,51,68].

## 5. Upstream Signaling to mTORC1

**Figure 2:**
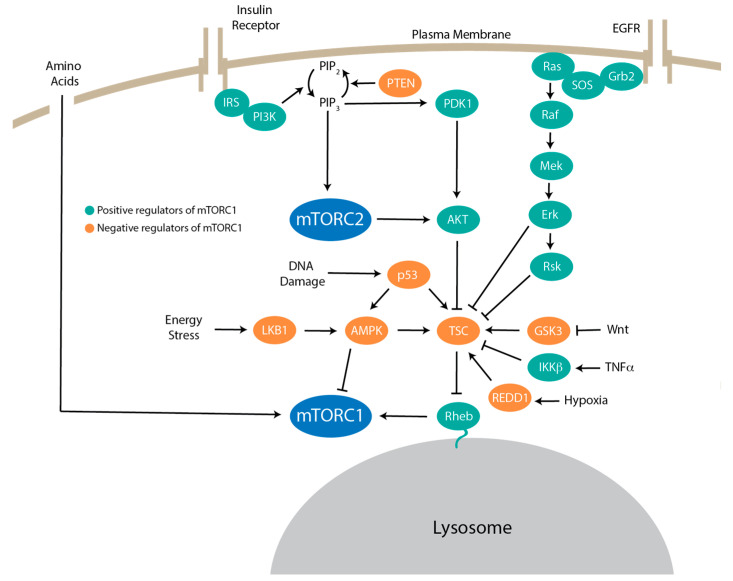
The mTOR upstream signaling network. Upstream regulators of mTOR signaling. Positive regulators of mTORC1 are shown in turquoise and negative regulators are shown in orange. Growth factors activate PI3K though the binding of IRS proteins. PI3K then phosphorylates PIP_2_ to PIP_3_ which then activates PDK1/2. Akt, containing a specific PIP_2_ and PIP_3_ PH domain, localizes to the plasma membrane and then subsequently activates through PDK1 phosphorylation. Akt promotes mTORC1 activity through the phosphorylation of TSC, subsequently activating Rheb. mTORC2 also phosphorylates Akt. The Ras-Raf-Mek-Erk signaling cascade leads to the inhibition of TSC through Erk or Rsk. mTORC1 activity is also controlled by Wnt signaling, TNFα through IKKβ, hypoxia through REDD1, and DNA damage through p53. Energy stress activates negative regulators such as LKB1 and AMPK to inhibit mTORC1. Rac-α Ser/Thr-protein kinase (Akt also known as PKB); AMP-activated protein kinase (AMPK); epidermal growth factor receptor (EGFR); extracellular signal-related kinase (Erk); GTPase activating protein (GAP); growth factor receptor-bound protein 2 (Grb2); glycogen synthase kinase 3 (GSK3); IκB kinase β (IKKβ); insulin receptor substrate (IRS); liver kinase B1 (LKB1); mitogen-activated protein kinase (MAPK); MAPK/ERK kinase (MEK); protein 53 (p53); pleckstrin homology (PH); phosphoinositide-dependent kinase 1/2 (PDK1/2); phosphoinositide 3-kinase (PI3K); phosphatidylinositol 4,5-bisphosphate (PIP_2_); phosphatidylinositol 3,4,5-triphosphate (PIP_3_); phosphatase and tensin homolog (PTEN); rapidly accelerated fibrosarcoma (Raf); rat sarcoma (Ras); DNA damage response 1 (REDD1); Ras homolog enriched in brain (Rheb); p90 ribosomal S6 kinase (Rsk); son of sevenless homolog (SOS); tumor necrosis factor α (TNFα); tuberous sclerosis complex (TSC); wingless-type (Wnt).

mTORC1 activation is controlled by growth factors, nutrients, energy status, and stress (Figure 2). Growth factor signaling regulates the tuberous sclerosis complex (TSC) [38]. Hamartin (TSC1), tuberin (TSC2), and Tre2-Bub2-cdc16 (TBC)1 domain family, member 7 (TBC1D7) comprise TSC [69]. TSC represses Rheb through its GTPase activating protein (GAP) activity, converting Rheb-guanosine triphosphate (GTP) to Rheb-guanosine diphosphate (GDP) [70,71]. Under TSC inhibition, Rheb is GTP bound, allowing binding and allosteric activation of mTORC1 through a conformational change [31,72]. Multiple upstream signals converge on TSC to regulate mTORC1 activity. For example, Akt [73], extracellular signal-regulated kinase (ERK) [74], p90 ribosomal S6 kinase 1 (RSK1) [75], IκB kinase β (IKKβ) [76], and MAPKAPK2 (MK2) [77] can phosphorylate and inhibit TSC2 [78] to activate mTORC1. Additionally, Wnt signaling can inhibit GSK3 phosphorylation of TSC2 to stimulate mTOR signaling. Other stimuli such as inflammation, hypoxia, low energy status, and DNA damage positively regulate the TSC complex to inhibit mTORC1 activity. Human diseases such as TSC and Lymphangioleiomyomatosis (LAM) result from mutations in the genes encoding TSC components, leaving mTORC1 in a constitutively active state [79,80]. TSC signaling represents an important signaling cascade that regulates mTORC1.

Low energy levels during glucose deprivation activate AMPK [81,82,83] through the phosphorylation of liver kinase B1 (LKB1) [84]. AMPK directly phosphorylates TSC2, increasing TSC2 GAP function [85]. In addition, AMPK phosphorylates mTORC1 component Raptor, increasing Raptor-14-3-3 binding and inhibiting mTORC1 [86]. During glucose deprivation, DNA damage response pathways lead to the inhibition of mTORC1 through the induction of tumor suppressor protein 53 (TP53) [87]. Other forms of stress, like hypoxia, result in mTORC1 inhibition. DNA damage and development 1 (REDD1) inhibits mTORC1 through TSC2, independently of AMPK [88,89].

## 6. Differential Regulation of mTORC1 by Amino Acids

Amino acids promote the lysosomal translocation and activation of mTORC1 [90,91]. Once at the lysosome mTORC1 becomes activated by Rheb GTP-bound downstream of growth factor signaling [92,93]. Thus, amino acids and growth factors are crucial in achieving optimal mTORC1 activity. mTORC1 activity is still sensitive to amino acid starvation in Tsc1 and Tsc2 knockout cells, demonstrating that growth factors and amino acids are distinct pathways that regulate mTORC1 activity [94,95,96].

**Figure 3:**
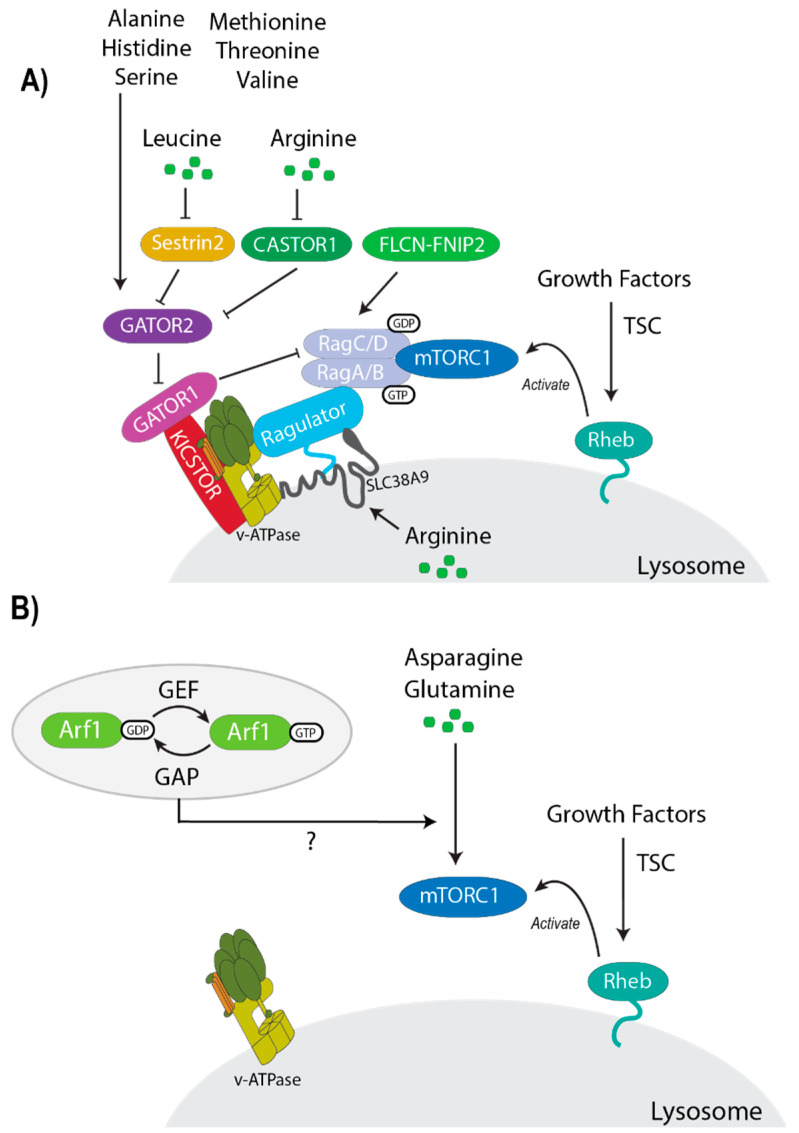
Amino acid sensing by mTORC1. (**A**) The Rag-dependent signaling pathway. Ala, Arg, His, Leu, Met, Ser, Thr, and Val can filter through upstream sensors in order to activate the Rag GTPases. Under sufficient amino acid conditions, the Rag GTPase heterodimer (GTP-RagA or RagB and GDP-RagC or RagD) interacts with mTORC1 at the lysosome, where Rheb resides. The Ragulator (consisting of p18, p14, MP1, C7orf59 and HBXIP) then anchors the Rag proteins to the lysosome and acts as a GEF for RagA and RagB. The FLCN-FNIP complex is a GAP for RagC and RagD. The v-ATPase, which is required for amino acid signaling to mTORC1, then binds to the Ragulator. KICSTOR (consisting of KPTN, ITFG2, C12orf66, and SZT2) anchors GATOR1 (consisting of DEPDC5, NPRL2 and NPRL3), the GAP for RagA and RagB, to the lysosome. GATOR2 (consisting of SEC13, SEH1L, WDR24, WDR59, and MIOS) inhibits GATOR1, through an unknown mechanism. Sestrin2 and CASTOR1 bind to GATOR2, preventing the inhibition of GATOR1 by GATOR2. Leu and Arg bind to sensors Sestrin2 and CASTOR1, respectively, which blocks Sestrin2-GATOR2 and CASTOR1-GATOR2 from interacting. (**B**) The Rag-independent signaling pathway. Only Gln and Asn activate mTORC1. The only known components that are required are the v-ATPase, Rheb, and the small GTPase Arf1. The cycling of Arf1 between a GTP- and a GDP-bound state promotes mTORC1 activation and lysosomal localization through an unknown mechanism. Adenosine diphosphate ribosylation factor 1 (Arf1); cellular Arg sensor for mTORC1 subunit 1 (CASTOR1); DEP domain containing 5 (DEPDC5); folliculin (FLCN); folliculin interacting protein (FNIP); GTPase-activating protein activity toward Rags (GATOR1); guanine nucleotide exchange factor (GEF); GTPase activating protein (GAP); hepatitis B virus X-interacting protein (HBXIP); integrin α FG-GAP repeat containing 2 (ITFG2); Kaptin (KPTN); meiosis regulator for oocyte development (MIOS); MEK partner 1 (MP1); mTOR complex 1 (mTORC1); NPR2 like, GATOR1 complex subunit (NPRL2); NPR3 like, GATOR1 complex subunit (NPRL3); vacuolar H+-ATPase (v-ATPase); Ras homolog enriched in brain (Rheb); SEH1-like nucleoporin (SEH1L); solute carrier family 38 member 9 (SLC38A9); seizure threshold 2 (SZT2); tuberous sclerosis complex (TSC); WD repeat-containing protein 24 (WDR24); WD repeat-containing protein 59 (WDR59).

The discovery of the Rag GTPases in 2008 advanced our understanding of how amino acid activate mTORC1 (Figure 3A) [90,97]. The Rag GTPase family of proteins consists of four members: RagA, RagB, RagC, and RagD [98]. RagA and RagB can form a heterodimer with RagC and RagD, with the possibility of forming four distinct complexes. RagA and RagB have high sequence similarity and are functionally redundant. Similarly, RagC and RagD are highly similar and also functionally redundant [98]. Deletion of RagA or RagB leads to the degradation of RagC and RagD, as the heterodimer stabilizes the Rag complex [90,99]. To activate mTORC1, RagA or RagB must be GTP bound while RagC/D is GDP bound. The active Rag GTPase complex binds to Raptor at the lysosome. The Ragulator complex is made up of p18, p14, MEK-binding partner 1 (MP1), chromosome 7 open reading frame 59 (C7orf59), and Hepatitis B virus X interacting protein (HBXIP) (LAMTOR 1-5, respectively). The Ragulator anchors the Rag complex and Raptor bound mTORC1 to the lysosomal surface through the N-terminal region of p18 [90,91,100,101]. Thus, the Rag GTPase-Ragulator complex plays an essential role in the recruitment and activation of mTORC1.

The guanine nucleotide loading of the Rag GTPases is controlled by guanine nucleotide exchange factors (GEFs) and GAPs. The Ragulator and solute carrier family 38 member 9 (SLC38A9) have been suggested to act as GEFs for RagA/B [100,102]. Ragulator GEF function and Rag GTPase-Ragulator binding depend on the lysosomal v-ATPase [103,104,105]. Amino acids that accumulate within the lysosomal lumen signal to the v-ATPase through an “inside out” mechanism of sensing, which in turn enhances GEF activity of the Ragulator. SLC38A9 functions as a GEF for RagA by stimulating GDP release from RagA upon activation specifically from Arg [102]. A trimeric complex known as GATOR1, consisting of DEP domain-containing 5 (DEPDC5), nitrogen permease regulator-like 2 (NPRL2), and nitrogen permease regulator-like 3 (NPRL3), acts as a GAP for RagA/B [106]. GATOR1 promotes RagA and RagB to hydrolyze GTP and inhibits mTORC1 during amino acid starvation conditions [107]. Upstream of GATOR1, GATOR2 positively regulates mTORC1 activity [106]. GATOR2, comprised of meiosis regulator for oocyte development (MIOS), WD repeat domain 24 (WDR24), WD repeat domain 59 (WDR59), Seh1 like nucleoporin (SEH1L) and Sec13 homolog nuclear pore and COPII coat complex component (SEC13), binds to and inhibits GATOR1 through an unclear mechanism. Another complex called KICSTOR is comprised of Kaptin (KPTN), integrin α FG-GAP repeat containing 2 (ITFG2), C12orf66 and seizure threshold 2 (SZT2), and tethers GATOR1 to the lysosome. GATOR1 requires KICSTOR to negatively regulate mTORC1 in response to amino acid deprivation [108,109]. Folliculin (FLCN) is a GAP for RagC or RagD. The folliculin interacting protein 2 (FLCN-FNIP2) complex GAP activity was shown to promote mTORC1-Rag heterodimer binding [110,111].

Some amino acid sensors for the Rag GTPase-dependent pathway have been identified. For example, two Arg sensors for mTORC1 have been identified for mTORC1, cellular Arg sensor for mTORC1 (CASTOR1) and SLC38A9 [112,113,114,115,116]. CASTOR1 homodimers or CASTOR1/2 heterodimer bind to and inhibit GATOR2 under Arg deprivation. CASTOR1 was shown to directly bind to Arg through the aspartate kinase, chorismate mutase, TyrA (ACT) domains. SLC38A9 interacts with the Ragulator, and cells lacking SLC38A9 have defective Arg-mediated mTORC1 activation. SLC38A9 has also been shown to play a role in mTORC1 activation by cholesterol [117]. Sestrin2 acts as a Leu sensor to inhibit GATOR2 during Leu withdrawal [118,119], and other amino acids like Met, Ile, and Val can disrupt the Sestrin2-GATOR2 interaction similar to Leu [120]. S-adenosylmethionine (SAM) sensor upstream of mTORC1 (SAMTOR) has been shown to be a sensor for a metabolite of Met. SAMTOR negatively regulates mTORC1 activity through the association with GATOR1 under Met deprivation [121]. The discovery of more sensors will reveal the differential regulation of mTORC1 by individual amino acids.

As mentioned above, SLC38A9 has been identified as part of the lysosomal machinery to control mTORC1. Other SLC transporters have been shown to regulate mTORC1 activity. For example, SLC15A4 was shown to transport His and oligopeptides out of the lysosome into the cytosol [122]. In B lymphocytes, loss of SLC15A4 impaired lysosomal acidification, and the study suggested the v-ATPase could be functionally or conformationally altered, negatively affecting mTORC1 activity. Another study showed that interleukin-18 (IL-18) enhanced the surface expression of the nutrient transporters CD98/LAT1 (encoded by slc3a2/slc7a5, respectively) in natural killer (NK) cells for amino acids to induce a metabolic change, such as enhanced glycolysis [123]. CD98/LAT1 is a bidirectional transporter for Leu and Gln [124,125]. Increasing the expression of the CD98/LAT1 heterodimer, also known as the System L transporter, enhanced mTORC1 activation by Leu. SLC3A2/SLC7A5, in addition to SLC1A5, have also been demonstrated to promote mTORC1 activity through Gln regulation [126]. SLC36A1 (PAT1), which transports Ala, Gly and Pro, was shown to positively regulate mTORC1 activation [127,128]. This transporter was found to drive mTORC1 signaling and contribute to cyclin dependent kinase 4/6 (CDK4/6) inhibitor resistance in melanoma. SLCs have been relatively understudied, and their involvement in amino acid signaling to mTORC1 should be a major focus of future research [129].

The Rag GTPase pathway was once considered the main mechanism for activation of mTORC1 by amino acids. However, it is now understood that a Rag GTPase-independent pathway exists, where Gln and Asn can activate mTORC1 (Figure 3B) [99]. In mammalian cells lacking the Rag GTPases, Gln was sufficient to stimulate mTORC1 lysosomal localization and activation. The activation of mTORC1 in this pathway required the v-ATPase and another small GTPase called adenosine diphosphate ribosylation factor 1 (Arf1) [99]. This was prefaced by a study in mouse cardiomyocytes demonstrating that a Cre-loxP RagA and RagB double conditional knockout mouse model retained mTORC1 activity [130]. Furthermore, Asn was found important for promoting mTORC1 activity in cancer cells [131]. Finding more signaling components and sensors will be important in order to further understand the Rag-independent pathway. Although, amino acids filtering through the Rag-dependent and Rag-independent pathways have been demonstrated to facilitate mTORC1 activation at the lysosome, the full mechanism of how each individual amino acid functions to stimulate mTORC1 remains largely unknown.

Leu, Arg, Met, and Gln have previously been shown to stimulate mTORC1 activity. However, a recent study from our group demonstrated that other amino acids are capable of activating mTORC1 (Table 1) [132]. Using mouse embryonic fibroblast (MEF) and human embryonic kidney 293A (HEK293A) cells, we found that 10 out of the 20 standard amino acids can activate mTORC1 in a time and dose dependent manner. Eight of these amino acids filter through the Rag-dependent pathway and have been involved in the regulation of mTORC1 activity (Ala [133,134], Arg [112,113,114,115,116,135], His [122], Leu [90,118,119], Met [121,133,136], Ser [133], Thr, and Val [133]). Ser, Thr, and Ala only induced a minor activation of mTORC1. These amino acids can be metabolized into pyruvate, which has been previously shown to positively regulate mTORC1 [137]. Moreover, it had been previously shown that some of these amino acids bind to known Rag-dependent sensors. Sestrin2 is a Leu sensor, but other amino acids (Met and Val) can disrupt the Sestrin2-GATOR2 interaction similar to Leu [120]. CASTOR1 is an Arg sensor and does not appear to bind to other amino acids [112]. Rag-dependent amino acids were also observed to induce mTORC1 activation quickly at ~15 min, whereas the Rag-independent amino acids took longer ~1 h for a peak in mTORC1 activity. Two amino acids filter through the Rag-independent pathway (Asn [131] and Gln [99,131,134,138,139]). In addition to activating mTORC1, surprisingly these ten amino acids induce mTORC1 lysosomal localization. Both the Rag-dependent and Rag-independent pathways require the v-ATPase and lysosomal function for the activation of mTORC1 [99,104,140]. Interestingly, Asn like Gln also requires the small GTPase Arf1 to activate mTORC1 [99]. Despite recent advances, there are many open questions remaining to how these specific amino acids stimulate mTORC1 activity.

With the advancement of cryo-electron microscopy (cryo-EM) technology, the structural understanding of components involved in amino acid signaling to mTORC1 have been better elucidated. Two different structures of mTORC1 with subunits mTOR, Raptor, and mLST8 were solved at a global resolution of 5.9 Å [141] and 4.4 Å [142]. These structures reveal how FKBP-rapamycin can inhibit some mTORC1 substrates from entering the catalytic cavity. Further studies of a 3.0 Å mTORC1 and a 3.4 Å structure of Rheb-mTORC1 demonstrate how Rheb causes global conformational change realigning active site residues within mTORC1 and allosterically activating mTORC1 [72]. Crystal structures of components in the amino acid sensing pathway such as GATOR1 alone or complexed with the Rag GTPases [107], Sestrin2 [118], and CASTOR1 [115] have also been solved. Recently, two reports built three-dimensional models of mTORC1 bound to Rheb, Rag GTPases and the Ragulator on the lysosomal surface [143,144]. Importantly, it demonstrates why only a specific nucleotide state of the Rag GTPases (RagA GTP-bound/RagC GDP-bound) allows binding of mTORC1. A newly identified region on Raptor referred to as the “Raptor claw,” further illustrated how Raptor and the Rag GTPases bind. The Raptor α-solenoid region detects the nucleotide state of RagA while the “claw” detects RagC. Structural findings like these illustrate a comprehensive understanding of the mTORC1 amino acid signaling pathway.

## 7. mTORC1 Regulation by GPCR Signaling

G-protein-coupled receptors (GPCRs) are seven-transmembrane pass proteins that are activated by an upstream ligand or agonist, resulting in the activation of multiple downstream signaling cascades [145]. GPCRs are highly conserved, and over 800 are encoded in the human genome [146]. GPCRs are coupled to the heterotrimeric G-proteins α, β and γ. Gα proteins are inactive in the GDP-bound state bound to β and γ. GPCRs function as a receptor-catalyzed GEF to activate Gα subunit and separate Gα from the Gβγ dimer through a conformational change. GTP hydrolyzing to GDP is the rate limiting step for Gα activity. The GDP-bound Gα subunit then rejoins the βγ dimer until the next activating cycle [147]. After Gα protein activation, the GPCR itself is phosphorylated by GPCR kinases (GRKs) followed by receptor internalization. This represents the classical view of GPCR signaling which occurs transiently at the cell surface followed by endocytosis [148]. There are currently four different Gα proteins: Gα_s,_ Gα_i/o_, Gα_q/11_ and Gα_12/13_ [149]. Gα_s_ and Gα_i/o_ regulate adenylate cyclases (ACs). Gα_s_ stimulates AC activity through binding, while Gα_i/o_ inhibits AC activity. Activation of ACs increase production and accumulation of intracellular 3′,5′-cyclic adenosine monophosphate (cAMP). Elevated cAMP levels regulate protein kinase A (PKA), exchange protein activated by cyclic AMP (EPAC), and cyclic-nucleotide-gated ion channels. Negative regulators of Gα_s_ signaling are cyclic nucleotide phosphodiesterases (PDEs), which can degrade cAMP [150]. Gα_q/11_ activates phospholipase C (PLC) and PLC can convert PIP2 to inositol 1,4,5-triphosphate (IP3) and diacylglycerol (DAG) [151]. IP3 gates a calcium channel in the ER to regulate cytoplasmic concentration of calcium. Furthermore, DAG is a substrate for synthesis of phosphatidic acid, which acts as a signaling lipid. Lastly, Gα_12/13_ targets GEFs for the small GTPase Ras homolog gene family (Rho), which has a role in actin cytoskeleton regulation [152]. Additional details on GPCR signaling can be found in other reviews [153,154].

**Figure 4:**
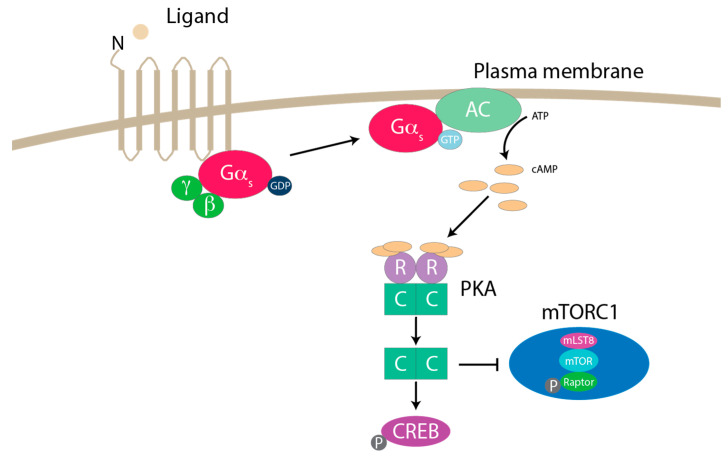
GPCR inhibition of mTORC1. Activation of Gα_s_-coupled GPCRs inhibit mTORC1 through the activation of PKA. The GPCR effector is a heterotrimeric G-protein made of three subunits (Gα, Gβ and Gγ). G-proteins are inactive in the GDP-bound state. GPCRs function as a receptor-catalyzed GEF to activate Gα subunit and separate them from the Gβγ dimer through conformational change. GTP hydrolyzing to GDP is the rate limiting step for Gα activity. Gα GDP-bound subunit then rejoins the βγ dimer until the next activating cycle. The GTP-Gα_s_ subunit can interact and turn on AC. AC is able to convert ATP cAMP. G-protein signaling induces cAMP, activating second messenger kinases such as PKA. PKA is a holoenzyme made of two regulatory subunits (R) and two catalytic subunits (C). R subunits have cAMP binding motifs. Two molecules of cAMP bind each R subunit to release and activate the C subunit of PKA. A well-known phosphorylation target of PKA is CREB at Ser 133. PKA can phosphorylate Raptor on Ser 791 to inhibit mTORC1 activity. adenylate cyclase (AC); 3′,5′-cyclic adenosine monophosphate (cAMP); G-protein coupled receptors (GPCRs); protein kinase A (PKA).

PKA is a Ser/Thr protein kinase holoenzyme that contains two catalytic and two regulatory domains [155]. The catalytic subunits release when two molecules of cAMP bind to each of the regulatory subunits [156]. Regulatory subunits (RI and RII) exist in either I/IIα or I/IIβ. RI subunits localize mainly to the cytoplasm, while RII subunits are found at membrane organelles [157,158]. Recently our lab uncovered the molecular mechanisms by which GPCRs-Gα_s_ signaling can regulate mTORC1 [159]. Specifically, we found that Gα_s_-coupled GPCRs increase cAMP to activate PKA and inhibit mTORC1 through the phosphorylation of Raptor on Ser 791 (Figure 4). One report showed that PKA phosphorylates Raptor on Ser 791 to promote mTORC1 activation in 3T3-L1 adipocytes [160]. However, a previous study had contrasting results showing that mTORC1 inhibition and active PKA signaling promote lipolysis in 3T3-L1 adipocytes [161]. Additional literature has demonstrated a relationship between cAMP signaling and mTORC1 activity. Interestingly, cAMP has been observed to either stimulate [162,163,164,165,166] or inhibit [167,168,169,170,171,172] mTORC1 activity, depending on the cell line experimentally used. For example, luteinizing hormone/human chorionic gonadotropin (LH/hCG) mediated activation of cAMP was shown to promote mTORC1 activity and increase primary theca-interstitial (T-I) cell proliferation [173]. In MEF and HEK293A cells however, the increase of cAMP by pharmacological activation inhibited mTORC1 activity through PKA [174]. One study in neurons showed that PKA and mTORC1 activity correlated in promoting protein synthesis [175]. Moreover, another report showed that in striatonigral medium spiny neurons (MSNs), activation of mTORC1 requires PKA signaling [176]. In human adrenocortical cells, PKA stimulation induced the activation of the mTOR pathway [177]. Similarly, signaling from the prostaglandin E2 receptor and subsequent activation of cAMP and PKA promoted mTORC1 activity through the phosphorylation of S6K1, S6, and 4EBP1 in pancreatic cancer (PANC-1) cells [178]. Thus, cAMP-PKA signaling can activate or inhibit mTORC1 depending on the cell type.

The broad expression of GPCRs makes them excellent therapeutic targets [179,180,181,182,183]. In 2017, 34% of all FDA-approved drugs targeted GPCRs [184]. Understanding how GPCR signaling regulates mTORC1 will be important for the potential repurposing of GPCR drugs to treat mTORC1 hyperactivation (Table 2). Initially, Gα_i/o_-coupled adrenergic receptor signaling was discovered to work through the PI3K pathway [185,186]. Additionally, mTOR-dependent vasopressin receptor signaling was shown to stimulate growth [187]. Studies of activated Gα_s_-coupled thyroid stimulating hormone [188], prostaglandin F2α [189], and orexin 1/2 receptors [190] led to an increase in mTORC1 activity through an Akt-independent pathway. Most of the understood crosstalk between GPCRs and mTORC1 occurred through the PI3K-Akt signaling pathway [191,192]. Evidence connecting the pathways (through adrenergic, muscarinic, and κ opioid receptors), lacked direct interaction [193,194,195,196]. GPCRs like Taste receptor type 1 member 1/3 (T1R1/T1R3) were demonstrated to sense amino acids, activate mTORC1 and inhibit autophagy [197]. Additionally, Gα_q/11_, Gα_s_, or Gα_i/o_-coupled GPCR signaling was found to regulate REDD1 and activate mTORC1 [198]. Recently, more direct evidence demonstrated GPCR regulation of mTORC1. Lysosomal localized GPCR-like protein (GPR137B) was shown to regulate amino acid-dependent mTORC1 activity through RagA/B [199]. Moreover, GPR137B was shown to interact with mTOR, Raptor and RagA. Understanding GPCRs and mTORC1 crosstalk will be crucial in targeting and inhibiting mTORC1.

The A-kinase anchoring protein (AKAP) family are scaffolding proteins that tether PKA to unique subcellular locations through the PKA regulatory domain [201]. AKAPs are not only compartmentalized, but also tissue specific, potentially making them valuable therapeutic targets and even biomarkers [155,202]. Phosphoproteomic studies have identified potential mTORC1 interacting AKAPs: AKAP1, AKAP2, AKAP8, AKAP8L, AKAP9, AKAP10, AKAP11, AKAP12, AKAP13, and AKAP28 using mass spectrometry [49,50,203]. Recently, our lab uncovered a novel interaction between mTORC1 and an AKAP [204]. We reported that AKAP8L could promote mTORC1 mediated processes such as protein translation, cell growth and proliferation. We also demonstrated that AKAP8L is not involved in the PKA inhibition of mTORC1 through phosphorylation of Raptor on Ser 791 [159,204]. Another finding showed that mitochondrial AKAP1 supports mTORC1 activation through binding and suppressing Sestrin2, a negative regulator of Leu signaling to mTORC1 [205]. The relationship among AKAPs and mTORC1 need to be further explored. Negative regulators of cAMP signaling like the PDEs, have also been shown to take part in mTORC1 signaling [166].

## 8. mTORC1 Phosphorylation and Regulation

mTORC1 activity is regulated through phosphorylation by multiple upstream kinases (Table 3). Studies have shown that Akt can phosphorylate mTOR at Thr 2446 and Ser 2448 promoting activity [206]. A later report demonstrated the in vitro phosphorylation of mTOR by Akt at those sites [207,208]. Subsequent studies revealed that S6K1 can also phosphorylate mTOR at Ser 2448 [209]. Another study showed that AMPK can phosphorylate mTOR at site Thr 2446 [210]. Ser 1261 on mTOR was found to be phosphorylated via PI3K signaling, promoting mTORC1 activity [211]. It was suggested that mTOR also has the capability of autophosphorylation at site Ser 2481, as exemplified in vivo and in vitro, and this phosphorylation site on mTORC1 correlates with intrinsic catalytic activity [212,213]. Downstream of Akt, IκB kinase α (IKKα) can phosphorylate mTOR at Ser 1415 promoting mTORC1 activity increasing cell proliferation in cancer cells [214]. Another group found that mTOR phosphorylation occurred on sites Ser 2159 and Thr 2164, through mass spectrometry and phopho-specific antibodies, promoting mTORC1 signaling [215]. They later demonstrated that TANK-binding kinase 1 (TBK1) interacts with and phosphorylates mTOR on Ser 2159, to promote catalytic activity of mTOR [216]. These studies provided more insight on how mTOR modulation occurs through phosphorylation.

Raptor is also phosphorylated on multiple sites. mTOR itself has been reported to phosphorylate Raptor in vitro and in vivo on Ser 863, where Ser 863 phosphorylation promotes mTORC1 activity [217]. In a subsequent study, clusters of Raptor phosphorylation sites were reported, including Ser 696, Thr 706, Ser 855, Ser 859, Ser 863, and Ser 877 [218]. Reaffirming previous studies, failure to phosphorylate Ser 863 resulted in lack of phosphorylation of Ser 859 and Ser 855. This study suggested a possible hierarchical phosphorylation to the sites on Raptor. Some known phosphorylation sites on Raptor still need further mechanistic understanding. Mechanical stimulus has been shown to induce the phosphorylation of Raptor on Ser 696, Thr 706, and Ser 863 resulting in mTORC1 activation [219]. Phosphorylation of these three sites also altered Raptor interaction with PRAS40 and S6K1, but not mTOR. RSK phosphorylation of Ser 719, Ser 721, and Ser 722 on Raptor positively regulates mTORC1 kinase activity [220]. This RSK mediated phosphorylation of Raptor was shown to be independent of the PI3K pathway. A follow up study found that ERK1/2 phosphorylates Raptor on sites Ser 8, Ser 696, and Ser 863 promoting mTORC1 activity [221]. Moreover, the phosphorylation of Raptor on these three sites did not regulate Raptor interaction with mTORC1 substrates. Raptor can also be the subject of osmotic stress-induced phosphorylation. c-Jun N-terminal kinase (JNK) directly interacts with and phosphorylates Raptor sites Ser 696, Thr 706 and Ser 863 in vitro [222,223]. JNK phosphorylation of Raptor positively regulates mTORC1 activity. Glycogen synthase kinase-3 (GSK3) has also been demonstrated to phosphorylate Raptor on site Ser 859, elevating mTORC1 activity [224]. GSK3 inhibition did not affect the lysosomal localization of mTORC1. However, inhibiting GSK3 did promote autophagic flux and reduced cell proliferation. During mitosis, cell division control 2 (cdc2) phosphorylates Raptor at Ser 696 and Thr 706 and is important for the promotion of mTORC1 activity and cell cycle progression [227,228].

Raptor also has multiple phosphorylation sites that lead to negative regulation. Raptor is a direct substrate of AMPK, with target sites on Ser 722 and Ser 792 [86]. This leads to phosphorylated-Raptor binding to 14-3-3 and inhibition of mTORC1. Another group found that Nemo-like kinase (NLK) phosphorylates Raptor on Ser 863, suppressing mTORC1 activation by disrupting lysosomal localization [225]. Hyperosmotic or oxidative stress activates NLK and inhibits mTORC1 through Rag GTPase-dependent signaling. NLK disrupts the interaction between Raptor and the Rag-GTPases. We and another group demonstrated Raptor can be phosphorylated by PKA on Ser 791, resulting in the regulation of mTORC1 [159,160]. Our report suggests that PKA phosphorylates Raptor on Ser 791 as a downstream result of GPCR-cAMP signaling, negatively regulating mTOR activity. Conversely, the other study found that PKA phosphorylation of Raptor on Ser 791 promotes mTOR activity. Recently, a group discovered the Hippo pathway component LATS1/2 kinase phosphorylate Raptor on Ser 606. [226]. LATS1/2 phosphorylation of Ser 606 induced suppression mTORC1 was suggested to result in a decreased organ size in mice. These studies represent how targeting a critical signal integrator for mTORC1 can have such a big impact. Interestingly, some sites, like Ser 863, have multiple modes of upstream regulation through different kinases which provides insight into how mTORC1 can be involved in many different signaling pathways.

## 9. Small GTPases that Regulate mTORC1

In the Rag-dependent amino acid (Ala, Arg, His, Leu, Met, Ser, Thr, and Val) signaling pathway to mTORC1, RagA/RagB and RagC/RagD form a heterodimer to shuttle mTORC1 to the lysosome [38]. In the Rag-independent amino acid (Asn and Gln) pathway, Arf1 is required for signaling to mTORC1 [99]. Though these small GTPases are essential in their respective pathways, there are other small GTPases that are implicated in mTORC1 regulation (Table 4). As briefly mentioned, the small GTPase Rheb is crucial for mTORC1 activation at the lysosome [38]. Rheb also localizes to other organelles including the ER, Golgi apparatus, peroxisome, and the mitochondria [229,230,231,232,233,234,235]. TSC serves as GAP for Rheb [70,71]. Akt [66,236], ERK [74], RSK1 [75], and IKKβ (IκB kinase β) [76] phosphorylate and inhibit TSC, which promotes Rheb bound to GTP and mTORC1 activation. In contrast, osmotic stress [237], AMPK [238], GSK3-β [239], REDD1 [88], and TP53 [87,240] have been shown to inhibit mTORC1 activity through TSC. A confirmed GEF for Rheb has yet to be identified. 

Ras-related protein Ral-A (RalA) has been shown to induce mTORC1 signaling, downstream of Rheb [241]. The study suggests that RalA has an important function in amino acid activation of mTORC1. RalA was previously reported to be involved in proliferation and oncogenic transformation [249,250]. Additionally, RalA has been attributed to anchorage-independent growth signaling through association with the exocyst complex, which is involved in vesicle transport [251] and glucose transporter type 4 (Glut4) translocation to the plasma membrane [252]. This relationship might shed light on the function of RalA in mTORC1 signaling, either through receptor localization or possibly insulin signaling [253].

The Ras-related protein Rab (Rab) family of GTPases have also been demonstrated to regulate mTORC1 activity. The Rab proteins are canonically known for their role in regulating the process of vesicular trafficking [242]. However, it was demonstrated that Rab5, Rab7, Rab10, and Rab31 potently inhibited mTORC1 activity [243]. This study indicated that proper regulation of intracellular trafficking is important for mTORC1 activity. Furthermore, Rab5 inhibits mTORC1 activation by amino acids through a Rag GTPase-dependent manner. Reports also present Rab12 as a regulator of mTORC1 activity. Through the control of amino-acid transporter proton-coupled amino acid transporter 4 (PAT4), Rab12 mediates mTORC1 activity and autophagy [244]. PAT4 indirectly modulates mTORC1 and subsequently autophagy through uptake of amino acids. Rab8A was found to modulate mTORC1 signaling in an immune response context [245]. Enriched on macrophage ruffles, Rab8A recruits PI3Kγ as an effector to regulate Akt signaling resulting from Toll-like receptor 4 (TLR4), a bacterial sensor for the innate immune response. This results in the activation of mTORC1 in response to the TLR4 pathogen recognition altering the cytokine response. Lastly, amino acid stimulation results in Rab1A-mTORC1 interaction on the Golgi, and overexpression of Rab1A promotes mTORC1 dependent oncogenic growth [233]. The Rab family presents an important field of study and broadens the interconnection of mTORC1 and other trafficking proteins.

The Rho GTPases are known for their function in cytoskeleton mediation, cell migration, proliferation, and transcription [254]. The three most studied members are Rho, Rac, and Cdc42. The main functions of these GTPases are to control stress fibers in addition to focal adhesion formation, regulate membrane ruffling and filopodium formation, respectively [254]. Previously, it was suggested that TSC1/2 activates Rac1 and inhibits Rho, through an unknown mechanism in the regulation of actin and focal adhesion remodeling [255]. Rac1 has also been identified as an mTOR binding protein. Rac1 regulates mTORC1 activity independently of PI3K and localizes mTORC1 at cellular membranes [246]. The absence of Rac1 inhibits mTORC1 activation and decreases cell size. Follow up work in mammalian cells by another group showed RhoA also suppresses mTORC1 kinase activity through an unknown mechanism upstream of Rheb [247]. Interestingly, RhoA did not interact directly with Raptor but did reduce the autophosphorylation of mTOR on Ser 2481.

A recent study reported that when amino acids are limited, Ras related protein 1 (Rap1) changes lysosome abundance which leads to the suppression of mTORC1 signaling [248]. Under amino acid limiting conditions, Rap1 concentrates at the lysosomes. Conversely, depletion of Rap1 leads to increased lysosome availability resulting in more interaction with mTORC1. It will be interesting to know what mechanistic detail future findings will reveal about this starvation response.

## 10. Conclusions, Future Directions, and Outstanding Questions

Research continues to uncover new components and molecular mechanisms of how growth factors and amino acids filter through upstream regulators in order to control mTORC1. Recent identification of new individual amino acids that regulate mTORC1 signaling could indicate the existence undiscovered upstream sensors involved in mTORC1 activation. Additionally, newly found amino acids that potently activate mTORC1 warrant further exploration. The identification of additional components involved in the Rag-independent pathway will provide more molecular insight to Gln- and Asn-induced mTORC1 activation. Why there are two distinct amino acid signaling pathways to mTORC1 and the difference in the signaling kinetics of these pathways to mTORC1 is currently not understood.

Understanding the mechanistic details of mTORC1 signaling provides major advances to the research community. However, we should not lose sight of the larger goal: Elucidating how mTORC1 signaling affects cellular physiology and how this pathway can be targeted for therapeutic benefit. The dysregulation of mTORC1 can be found in many diseases ranging from diabetes, cancer, neurodegeneration and so forth [38]. Revealing how GPCR signaling regulates mTORC1 activity could help redefine the way we target mTORC1 clinically. For example, in 2017 approximately 9% of total therapeutics in clinical trials for diabetes were against GPCRs [184]. In fact, 11 GPCRs were involved in approved treatments, while 25 GPCR targeting drugs were under clinical trial at the time of the publication. The pursuit of treating type 2 diabetes has stimulated further interest in GPCR therapeutics [256,257]. The Glucagon-like peptide 1 (GLP-1) receptor agonist, exenatide, was the first GPCR targeted drug for type 2 diabetes in 2005, and several more GLP1 receptor agonists have been approved since. A recent study showed that GLP-1 receptor signaling involved mTOR [200], demonstrating that targeting GPCRs could have benefits in diabetic therapies with dysregulated mTORC1 signaling [258]. Other mTOR associated diseases, such as cancer, have also proved to be opportunistic for GPCR drug targets [259], as over 20 agents were in clinical trials at the time of that publication. Studying components of the GPCR-Gα_s_ pathway, such as AKAPs, PKA regulatory subunits, and phosphodiesterases involved in mTORC1 regulation, would also be extremely beneficial. For example, PKA phosphorylation of Ser 791 on Raptor leads to activation or inhibition of mTORC1 depending on the cell type. Therefore, utilizing FDA approved drugs (agonists and antagonists) that target GPCR-Gα_s_, could modulate mTORC1 activity. Different tissues contain mixtures of GPCR populations [179,180]. Repurposing GPCR drugs for new indications could prove advantageous as about 33% of these drugs are known to have multiple uses as of 2017 [184]. Furthermore, there is potential use for treatment of mTOR related diseases that are either tissue specific or require multiple therapeutic approaches [38]. The failures of rapamycin due to the inadequacy of complete inhibition of mTORC1 leaves a desperate need for a better solution. Exploring mTORC1 regulation through GPCR signaling might provide an alternative method.

mTORC1 phosphorylation has provided more insight into the mechanism of mTORC1 regulation by upstream stimuli. Furthermore, studying the small GTPases involved in mTORC1 regulation contribute to another important area of research. Lysosomal translocation and activation of mTORC1 can be regulated by the Rag GTPases. However, the localization of mTORC1 at the lysosome stems multiple questions. Where are mTORC1 substrates phosphorylated? Some known substrates are not localized to the lysosome. How is mTORC1 trafficked to the lysosome, and where is mTORC1 located when it’s not at the lysosome? One possibility could be the regulation of mTORC1 by other small GTPases, many of which are associated with cellular trafficking. Additionally, other small GTPases could regulate mTORC1 at different subcellular locations. How these small GTPases function in accordance with amino acid and growth factor signaling will provide more insight into mTORC1 regulation.

## Figures and Tables

**Table 1:** Amino acids known to activate mTORC1.

Amino Acid	Rag GTPase	Sensor	Reference
Alanine	Dependent	?	[132,133,134]
Arginine	Dependent	CASTOR1, SLC38A9	[112,113,114,115,116,132,135]
Asparagine	Independent	?	[131,132]
Glutamine	Independent	?	[99,131,132,134,138,139]
Histidine	Dependent	?	[122,132]
Leucine	Dependent	Sestrin2	[90,99,118,119,132,133]
Methionine	Dependent	SAMTOR	[112,121,132,133]
Serine	Dependent	?	[132]
Threonine	Dependent	?	[132]
Valine	Dependent	?	[132,133]

? = Unknown; Amino acids and their corresponding signaling pathway are described. Only some have known upstream sensors. Ala, Arg, Asn, Gln, His, Leu, Met, Ser, Thr, and Val can all shuttle and promote mTORC1 activation. Other amino acids (Met, Ile, Val) have been shown to disrupt the Sestrin2-GATOR2 interaction similar to Leu. Cellular Arg sensor for mTORC1 subunit 1 (CASTOR1); solute carrier family 38 member 9 (SLC38A9); S-adenosylmethionine [SAM] sensor upstream of mTORC1, or C7orf60 (SAMTOR).

**Table 2:** GPCRs known to regulate mTORC1 activity.

GPCR	Coupled G-protein	mTORC1 Activity	Model/Cell Line	Reference
GLP-1	Gα(s)	Increase	BRIN-BD11	[200]
α2-AR	Gα(i/o)	Increase	PC-12	[185]
M_4_mAChR	Gα(i/o)	Increase	PC-12	[186]
V_1_	Gα(s)	Increase	Rat mesangial cells	[187]
TSH	Gα(s)	Increase	Rat thyroid, CHO	[188]
PGF2α	Gα(s)	Increase	bLCs	[189]
OX1/2R	Gα(s)	Increase	HEK-293T, N41, MEF	[190]
mGluR	Gα(i/o)	Increase	Primary neuronal	[193]
KOR	Gα(i/o)	Increase	Adult male CD-1 mice, N2A-FmK6H cells	[196]
T1R1/T1R3	Gα(i/o)	Increase	MIN6	[197]
GPR137B	?	Increase	Hs68, HeLa, HEK-293T/E, HAP1, MEF, zebrafish	[199]
β1/β2-AR, AVP, GCGR	Gα(s)	Decrease	Various cell lines, Primary hepatocytes, mice	[159]

? = Unknown; Listed are some of the known GPCRs that positively or negatively regulate mTORC1. Glucagon-like peptide 1 (GLP-1); α-2 adrenergic receptor (α2-AR); Muscarinic acetylcholine receptor M4 (M_4_ mAChR); Vasopressin V1 receptor (V_1_); Thyroid stimulating hormone receptor (TSH); Prostaglandin F2-α receptor (PGF2α); Orexin 1/2 receptor (OX1/2R); Metabotropic glutamate receptor (mGluR); kappa opioid receptor (KOR); Taste receptor type 1 member 1/3 (T1R1/T1R3); GPCR 137B (GPR137B); β-1/β-2 adrenergic receptor (b1/b2-AR); Arg vasopressin receptor (AVP); Glucagon receptor (GCGR). Rat pancreatic hybrid cells of NEDH and RINm5F (BRIN-BD11); rat adrenal gland cells (PC-12); Chinese hamster ovary cells (CHO); bovine steroidogenic luteal cells (bLCs); Human embryonic kidney 293T cells (HEK-293T); mouse embryonic hypothalamus N41 cells (N41); Mouse embryonic fibroblast (MEF); mouse neuro2A neuroblastoma with FmK6H construct (N2A-FmK6H); mouse insulinoma 6 (MIN6) human primary fibroblasts (Hs68); Henrietta Lacks cervical adenocarcinoma cells (HeLa); haploid 1 cells (HAP1). G-proteins presumed, not all GPCRs were presented with coupled Gα protein in respective reference.

**Table 3:** Known phosphorylation sites of mTORC1.

mTORC1 Subunit	Site Phosphorylated	Kinase	mTORC1 Activity	Reference
mTOR	Thr 2446, Ser 2448	Akt	Increase	[206,207,208]
	Ser 2448	S6K1	Increase	[209]
	Thr 2446	AMPK	Decrease	[210]
	Ser 1261	?	Increase	[211]
	Ser 2481	mTOR	Increase	[212,213]
	Ser 1415	IKKα	Increase	[214]
	Ser 2159, Thr 2164	TBK1, ?	Increase	[215,216]
Raptor	Ser 863, Ser 859	mTOR	Increase	[217,218]
	Ser 696, Thr 706, Ser 863	?	Increase	[219]
	Ser 719, Ser 721, Ser 722	RSK1/2	Increase	[220]
	Ser 8, Ser 696, Ser 863	ERK1/2	Increase	[221]
	Ser 696, Thr 706, Ser 863	JNK	Increase	[222,223]
	Ser 859	GSK3	Increase	[224]
	Ser 722, Ser 792	AMPK	Decrease	[86]
	Ser 863	NLK	Decrease	[225]
	Ser 791	PKA	Decrease	[159,160]
	Ser 606	LATS1/2	Decrease	[226]
	Ser 696, Thr 706	cdc2	Increase	[227,228]

? = Unknown; mTORC1 components mTOR and Raptor and the corresponding phosphorylation sites, kinase and effect on mTORC1 activity. Rac-α Ser/Thr-protein kinase (Akt also known as PKB); p70 ribosomal S6 kinase 1 (S6K1); AMP-activated protein kinase (AMPK); mammalian or mechanistic target of rapamycin (mTOR); IκB kinase α (IKKα); TANK-binding kinase 1 (TBK1); p90 ribosomal S6 kinase 1/2 (Rsk1/2); extracellular signal-related kinase 1/2 (Erk1/2); c-Jun N-terminal kinase (JNK); glycogen synthase kinase 3 (GSK3); Nemo-like kinase (NLK); protein kinase A (PKA); Large tumor suppressor kinase 1/2 (LATS1/2); cell division control 2 (cdc2).

**Table 4:** Small GTPases known to regulate mTORC1.

Small GTPase	mTORC1 Activity	Reference
RagA/B, RagC/D	Increase	[38]
Rheb	Increase	[38,70,72]
RalA	Increase	[241]
Rab5	Decrease	[242,243]
Rab12	Decrease	[244]
Rab8a	Increase	[245]
Rab1a	Increase	[233]
Rac1	Increase	[246]
RhoA	Decrease	[247]
Rap1	Decrease	[248]

Listed are known small GTPases and their effect on mTORC1 activity. Ras related GTP binding protein (Rag); Ras homolog enriched in brain (Rheb); Ras-related protein Ral (Ral); Ras-related protein Rab (Rab); Rho homolog gene family (Rac); Ras homolog gene family (Rho); Ras-related protein (Rap).
